# Dietary Polyunsaturated Fatty Acids and Inflammation: The Role of Phospholipid Biosynthesis

**DOI:** 10.3390/ijms141021167

**Published:** 2013-10-22

**Authors:** William Raphael, Lorraine M. Sordillo

**Affiliations:** Department of Large Animal Clinical Sciences, College of Veterinary Medicine, Michigan State University, 736 Wilson Rd., Room D202, East Lansing, MI 48824, USA; E-Mail: raphaelw@msu.edu

**Keywords:** diet, phospholipid, fatty acid, lipid mediator, eicosanoid, inflammation, lipoxygenase, cyclooxygenase

## Abstract

The composition of fatty acids in the diets of both human and domestic animal species can regulate inflammation through the biosynthesis of potent lipid mediators. The substrates for lipid mediator biosynthesis are derived primarily from membrane phospholipids and reflect dietary fatty acid intake. Inflammation can be exacerbated with intake of certain dietary fatty acids, such as some ω-6 polyunsaturated fatty acids (PUFA), and subsequent incorporation into membrane phospholipids. Inflammation, however, can be resolved with ingestion of other fatty acids, such as ω-3 PUFA. The influence of dietary PUFA on phospholipid composition is influenced by factors that control phospholipid biosynthesis within cellular membranes, such as preferential incorporation of some fatty acids, competition between newly ingested PUFA and fatty acids released from stores such as adipose, and the impacts of carbohydrate metabolism and physiological state. The objective of this review is to explain these factors as potential obstacles to manipulating PUFA composition of tissue phospholipids by specific dietary fatty acids. A better understanding of the factors that influence how dietary fatty acids can be incorporated into phospholipids may lead to nutritional intervention strategies that optimize health.

## 1. Introduction

Dietary fatty acids can control the incidence and severity of inflammation in some diseases of humans and domestic animals. Current evidence suggests that the dietary ω-6 (n-6) to ω-3 (n-3) polyunsaturated fatty acid (PUFA) ratio is directly associated with inflammatory-based pathology in human diseases such as cancer, rheumatoid arthritis, atherosclerosis, and obesity [1–3]. Studies in production animals used for human food also suggest that dietary intake of certain PUFA can impact inflammatory responses to common infectious diseases such as mastitis and metritis [4,5]. Associations between dietary fatty acids and diseases are partially explained by the incorporation of dietary n-6 and n-3 PUFA into membrane phospholipids [6]. The PUFA content of phospholipids influence inflammation through several mechanisms including membrane fluidity, lipid raft formation, and receptor function [7,8], but the focus of this review is on the biosynthesis of potent phospholipid-derived lipid mediators that have pro- or anti-inflammatory functions. For example, many lipid mediators produced from n-6 PUFA have pro-inflammatory functions and the overexpression of these lipid mediators is associated with the pathogenesis of inflammation during disease. Conversely, lipid mediators derived from n-3 PUFA have inflammation-resolving properties, and partly explain the disease-protective effects that are sometimes seen with n-3 PUFA ingestion [9]. Inconsistent health benefits seen with n-3 PUFA supplementation could be due to the regulation of dietary and stored PUFA incorporation into phospholipids. Digestion, for example, can change the profile of ingested fatty acids by reducing the number of double carbon bonds [10,11]. Additionally, fatty acid incorporation into phospholipids is sometimes selective [12]. Also, phospholipid synthetic pathways are regulated at multiple levels and are sometimes controlled by other nutrients or metabolites, such as diacylglycerol and glucose [13,14]. The objectives of this review are to explain how dietary n-6 and n-3 PUFA are incorporated into membrane phospholipids, explain how the subsequent biosynthesis of lipid mediators can influence inflammatory responses, and describe factors confounding the influence of dietary PUFA on phospholipid composition. Future studies of these confounding factors will improve knowledge of phospholipid biosynthesis in humans and domestic animals and will reveal all health outcomes of n-3 PUFA dietary supplements.

## 2. Trends in Dietary Fatty Acid Composition

The consumption of n-3 and n-6 PUFA in Western diets changed considerably over the last several decades [15]. The n-6 PUFA content of Western human diets increased to some extent because plants or plant-derivatives, such as corn oil, are popular dietary ingredients [16]. In contrast to modern Western human diets, Northern (e.g., Greenland) and Eastern (e.g., Southeast Asian) diets contain more n-3 PUFA because proportionally more dietary fatty acid is derived from fish [17]. Marine plants and fish are rich in n-3 PUFA, relative to terrestrial plant- or animal-derived foodstuffs that are abundant in Western diets. Hence the dietary n-6 to n-3 PUFA ratio is higher in modern Western human diets compared to regions where fish remain a significant source of PUFA.

In spite of these differences in PUFA intake between regions, both n-6 and n-3 remain important dietary ingredients in all human and domestic animals. Linoleic acid (LA, C18:2 n-6) nutritional deficiency results in disease of multiple systems, including skin [18] and α-linolenic acid (ALA, C18:3 n-3) deficiency results in neurological impairment [19]. These PUFA cannot be synthesized *de novo* but, once absorbed, they can be converted to other PUFA within the same omega designation [20]. Domestic animals and humans cannot synthesize n-3 PUFA from n-6 because they lack specific desaturase enzymes, such as that transcribed from *fat-1* in lower order eukaryotes [3]. Therefore, the n-6 to n-3 PUFA ratio in tissue phospholipids is a direct reflection of dietary composition, although the profiles of individual n-6 and n-3 PUFA are also subject to *de novo* elongation and desaturation [20].

Like humans, many species of domestic animals have experienced significant dietary change over the past several decades. This is partly due to industrialization of Western agriculture, which reduced or eliminated pasture feeding and introduced concentrated-nutrient forages into the diet of many species, including ruminants. Meat and milk derived from intensively-raised ruminants may be partly responsible for elevated n-6 to n-3 PUFA ratios in Western human diets [16] as modern ruminant diets were shown to affect the n-6 to n-3 PUFA ratio of meat [21] and milk [22] from these animals. As with humans, there is increased disease risk in ruminants fed these modern diets [23], which can be partly explained by the n-6 to n-3 PUFA ratio in phospholipids and the effect this has on severity and duration of host inflammatory responses [24].

## 3. Dietary Polyunsaturated Fatty Acids and Inflammation

Inflammation is an essential component of the innate immune response to tissue injury. Movement of serum proteins, lipids, and blood leukocytes into affected tissues eliminate or neutralize the source of tissue injury then restore normal tissue structure and function. Failure to control the magnitude and duration of the inflammatory response can cause damage to host tissues and contribute to pathology, independent of the original insult. The ratio of n-6 to n-3 PUFA in the diet has long been recognized as an important influence on the pathogenesis of inflammation because PUFA derivatives can initiate and exacerbate inflammatory responses [1]. This was demonstrated in several human and animal diseases, including atherosclerosis, sepsis, mastitis, and cancer [3,4,25], and involves derivatives of n-6 PUFA. In contrast, human diseases with inflammatory-based pathology were prevented with n-3 PUFA dietary supplementation. Increased fish-derived n-3 PUFA consumption, for example, was shown to lower risk of cardiovascular diseases and improved survival from myocardial infarction [26–28]. Evidence also exists that dietary n-3 PUFA dietary supplementation can prevent several forms of cancer [29]. The inflammatory response of healthy human subjects is also affected by n-3 PUFA ingestion. For example, human mononuclear leukocytes from subjects supplemented with dietary fish-oil have decreased TNF-α expression relative to subjects consuming control diets [30]. Despite this evidence, there remains some controversy concerning the health benefits of n-3 PUFA ingestion because not all data were conclusive. Dietary supplementation with 1 g of n-3 PUFA daily, for example, did not reduce the rate of adverse cardiovascular events in patients that had or were at risk of type 2 diabetes [31]. Also a recent meta-study found no association between n-3 PUFA intake and incidence of major cardiovascular disease [32]. A better understanding of how dietary PUFA are integrated into membrane phospholipids and therefore available for biosynthesis of pro-resolving mediators of inflammation may explain these equivocal results.

### 3.1. Phospholipids of Inflammatory Cells

**Figure 1:**
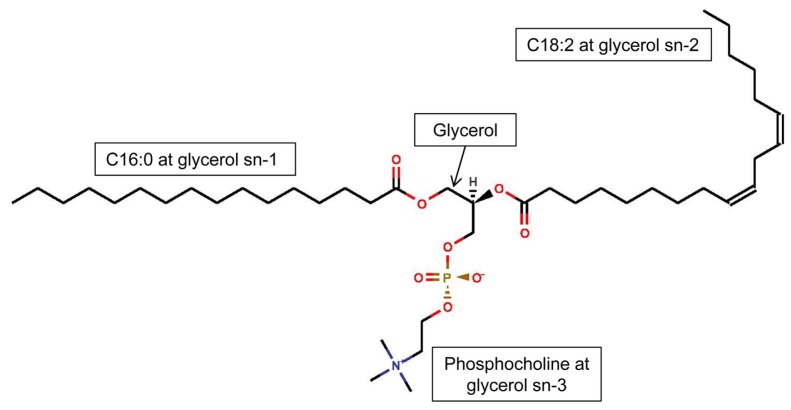
Phospholipids consist of glycerol, usually esterified to a saturated long chain fatty acid at sn-1 and to an unsaturated long chain fatty acid at sn-2, and to a phosphorylated head group at sn-3. This example is 1-hexadecanoyl-2-(9*Z*,12*Z*-octadecadienoyl)-*sn-*glycero- 3-phosphocholine.

The link between dietary PUFA, inflammation, and disease susceptibility is partially due to changes in the PUFA content of phospholipids in cells involved in the inflammatory response, such as monocytes, macrophages, and vascular endothelial cells [33,34]. Phospholipids are composed of 2 fatty acids esterified to glycerol at sn-1 and sn-2 and a phosphorylated head group esterified at sn-3 (Figure 1). The n-6 and n-3 PUFA, including arachidonic acid (AA, C20:4 n-6), LA, eicosapentaenoic acid (EPA, C20:5 n-3) and docosahexaenoic acid (DHA, C22:6 n-3) are incorporated into the sn-2 position of phospholipids in cellular membranes [12]. Phospholipid head groups are cytidine-monophosphate, hydroxyl, choline, ethanolamine, serine, or inositols [12]. Phosphocholine and phosphoethanolamine are concentrated in macrophage plasma membranes [35], are the major phospholipids in human plasma (76% phosphocholine and 17% phosphoethanolamine) [36], and are also the most abundant phospholipids in human erythrocytes [37]. As such, phosphocholine and phosphoethanolamine are the largest phospholipid reservoirs of dietary n-6 and n-3 PUFA in cells and fluids involved in inflammatory responses.

Other less abundant phospholipids can affect inflammatory responses, but independent of lipid mediator production. For example, diphosphoglycerol (cardiolipin) consists of 2 molecules of phosphatidic acid esterified to glycerol, is the most abundant phospholipid in the inner mitochondrial membrane [38], and can compromise cellular respiration [39] and mediate apoptosis [40] under conditions of oxidative stress. Another example is platelet activating factor (1-*O*-alkyl-2-acetyl-*sn*-glycero-3-phosphocholine), which directly enhances cytokine and adhesion molecules expression in vascular endothelium [41]. These are important effects of phospholipids but the objective here is to explain how major phospholipids in cells and fluids that are critical in inflammatory processes are influenced by dietary PUFA and regulate inflammatory processes when PUFAs are converted to lipid mediators of inflammation.

### 3.2. Inflammatory Pathways Influenced by Fatty Acid Components of Phospholipids

The fatty acid composition of membrane phospholipids in macrophages and endothelium can influence inflammatory responses in several different ways. Physical properties of membranes, such as fluidity and lipid raft formation, are influenced by specific fatty acids in membrane phospholipids and modify membrane-generated signaling cascades. For example, murine macrophage and adipocyte and human vascular endothelial cell culture studies, including some with transgenic mice, demonstrated that saturated fatty acids can directly activate proinflammatory signaling pathways through Toll-like receptor (TLR)-mediated mechanisms [42–44]. Saturated fatty acids increase proinflammatory gene expression in macrophages by inducing the dimerization and recruitment of TLR4-related signaling proteins into lipid rafts in a way that mimics endotoxin [42,45]. These responses to saturated fatty acids in phospholipids involve downstream pro-inflammatory signaling pathways. For example, treatment of murine macrophage cell lines with palmitic (C16:0) and lauric (C12:0) acids increased phosphorylation of JNK and ERK, resulting in enhanced expression of proinflammatory cytokines [45]. Interestingly, the saturated fatty acid-induced increase in phosphorylation of MAPK signaling subunits was attenuated by increasing the DHA content of cells [45]. This effect may be partially attributable to the direct action of n-3 PUFA and independent of conversion to lipid mediators [46,47]. Although saturated fatty acids and PUFA have important direct effects on health, the focus of this review is on conversion of n-3 and n-6 PUFA, derived from membrane phospholipids, into the more potent lipid mediators [47].

### 3.3. Phospholipid-Dependent Biosynthesis of Lipid Mediators

**Figure 2:**
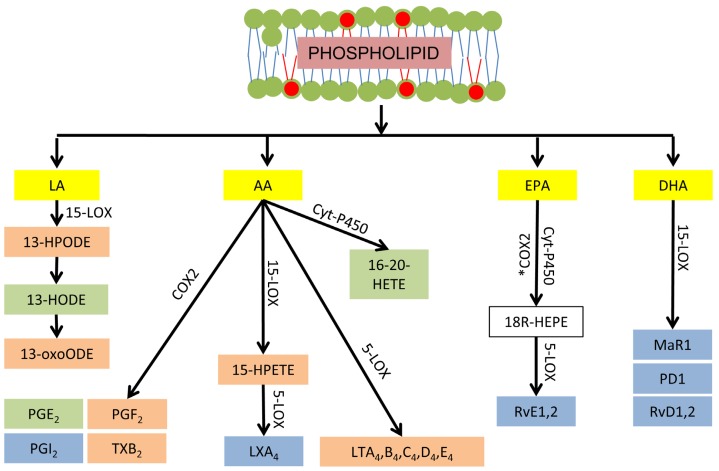
Fatty acyl chains of phospholipids located within the membranes of cells and organelles may be enzymatically oxidized into lipid mediators. Examples of proinflammatory (**orange**), resolving (**blue**), and variable function (**green**) lipid mediators are illustrated by fatty acid substrate (**yellow**) and biosynthetic enzyme. *: aspirin acetylated; LA: linoleic acid; AA: arachidonic acid; EPA: eicosapentaenoic acid; DHA: docosahexaenoic acid; COX: cyclooxygenase; Cyt-P450: cytochrome-P450 complex; HEPE: hydroxyeicosapentaenoic acid; HETE: hydroxyeicosatetraenoic acid; HODE: hydroxyoctadecadienoic acid; LOX: lipoxygenase; LT: leukotriene; LX: lipoxin; MaR: maresin; PG: prostaglandin; Rv: resolvin; PD: protectin; TX: thromboxane.

Several hundred lipid mediators have been identified and they collectively regulate the initiation, magnitude and duration of inflammatory responses [48]. Eicosanoids are the most widely studied class of lipid mediators. These are derived from AA and include hydroperoxyeicosatetraenoic acids (HPETE), prostaglandins (PG), thromboxanes (TX), leukotrienes (LT), and lipoxins. The first step in eicosanoid biosynthesis occurs when esterified AA is released from membrane phospholipids by phospholipase enzymes. Intracellular AA may then be metabolized by the cyclooxygenase (COX), lipoxygenase (LOX), or epoxygenase (e.g., cytochrome P450) enzymatic pathways, which ultimately determine the class of eicosanoid generated (Figure 2). Two isoforms of COX enzymes, denoted as COX1 and COX2, are involved in the enzymatic oxidation pathways. Both COX isoforms first catalyze the oxidation of AA to prostaglandin G_2_ (PGG_2_) followed by a peroxidase reaction that reduces PGG_2_ to PGH_2_[49]. From PGH_2_, specific downstream PG synthases produce PGE_2_, PGD_2_, PGI_2_ and PGF_2α_. Alternatively, thromboxane synthases convert PGH_2_ to TXA_2_ and TXB_2_. Similar to the COX family, there are several isoforms of LOX involved in the enzymatic oxidation of PUFA. For example, 5-LOX catalyzes the oxidation of AA to 5-HPETE that can be further metabolized to produce leukotrienes (LT). Moreover, both 15-LOX1 and 15-LOX2 oxidize AA to 15-HPETE [50]. This can be further metabolized to lipoxin A_4_ by 5-LOX [51]. The eicosanoid class of lipid mediators is predominantly proinflammatory, with a small number of exceptions. For example, lipoxin A_4_ is pro-resolving [51] and PGE_2_ has both proinflammatory and resolving effects [52]. Whereas AA is the common precursor of the eicosanoid class of lipid mediators, there are other important mediators produced from the enzymatic oxidation of LA, DHA, and EPA. For example, LA is metabolized by several enzymes including 15-LOX, COX2, and the epoxygenases into hydroperoxyoctadecadienoic acid (HPODE). This is reduced to hydroxyoctadecadienoic acid (HODE), which is dehydrogenated to form oxooctadecadienoic acid (OxoODE) [51]. The effects of LA derived mediators can be either proor anti-inflammatory [53–55]. Lipid mediators derived from n-3 PUFA are the most recently discovered [51]. These mediators are synthesized from DHA by 12-LOX or 15-LOX and are referred to as resolvins, protectins, and maresin. In contrast to the mediators derived from n-6 PUFA, the function of mediators derived from n-3 PUFA are primarily anti-inflammatory or pro-resolving [51]. COX2 also synthesizes resolvins from EPA, but only in patients treated with aspirin (Figure 2). Alternatively, these EPA derived resolvins can be synthesized by epoxygenases [51].

Enzymatic biosynthesis of lipid mediators is important because enzymes can be overexpressed in diseases such as atherosclerosis and sepsis [46,56]. Both n-6 and n-3 PUFA, however, may also undergo non-enzymatic oxidation with reactive oxygen species, resulting in production of lipid mediators with similar biological effects to those derived from enzymatic oxygenation [57]. For example, some 15-HPETE and 13-HPODE isomers produced by non-enzymatic oxidation are identical to those synthesized by enzymatic oxidation [58,59]. These hydroperoxides are themselves reactive oxygen species, thus creating positive feedback loops on PUFA oxidation during an inflammatory response that can exacerbate disease pathogenesis.

### 3.4. Dietary PUFA Influence Inflammation through Lipid Mediators

There is strong evidence that the dietary n-6 to n-3 PUFA ratio influences the profile of lipid mediators within tissue [9,60,61]. For example, a recent human study demonstrated increased plasma HODE and OxoODE with increased dietary LA [60]. Dietary supplementation with AA increased mononuclear leukocyte secretion of pro-inflammatory eicosanoids, including LTB_4_[61]. EPA and DHA dietary supplementation were shown to increase lipoxins and E and D series resolvins in mouse tissue, while also decreasing pro-inflammatory AA-derived eicosanoids [9]. Therefore *in vivo* production of lipid mediators is regulated by substrate availability. Some of these studies have confirmed that dietary n-6 to n-3 PUFA ratio does in fact influence lipid mediator biosynthesis through changes in PUFA composition of tissue phospholipids [9,62].

Manipulation of the profile of tissue lipid mediators by dietary PUFA is significant because lipid mediators are known to influence health. For example, increased AA-derived PGE_2_ biosynthesis is associated with human colon cancer [63,64]. There is increased expression of LA-derived metabolites, 9-HODE and 13-HODE, in atherosclerotic vascular lesions [65]. Specific effects of eicosanoids on inflammation and disease pathogenesis have been demonstrated using *in vivo*, *in vitro*, and transgenic animal models of disease. For example, DHA-derived resolvin D2 was shown to ameliorate the inflammatory response in murine models of sepsis [66] and blockade of resolvin receptors in human polymorphonuclear leukocytes resulted in loss of the anti-inflammatory effects of resolvin D1 [67]. PGE_2_ receptor deletion reduced pathology in murine models of colon cancer [64]. Several studies showed that deletion of the *12/15-LOX* gene was effective in decreasing atherosclerosis pathology in mice [68,69].

Despite cumulative evidence that changes in the profiles of eicosanoids and other lipid mediators are a major factor contributing to the health effects of PUFA intake, there are several important studies that are inconclusive. For example, a recent review of 15 human LA dietary trials found no association between intake and pro-inflammatory markers, including eicosanoids and other lipid mediators [70]. A recent meta-analysis of the protective effects of n-3 PUFA supplementation against human cardiovascular diseases failed to identify any significant health benefits [32]. There are several possible reasons why such inconsistency may exist in the literature. For example, lipid mediator biosynthesis is regulated at multiple levels in addition to supply of PUFA substrate. These include regulation of enzyme transcript and protein expression and enzyme activity [56,71–76]. Also, some n-6 PUFA are substrate for pro-resolving mediators, which can be displaced by n-3 PUFA supplementation [9]. Additionally, enzymes that synthesize lipid mediators are selective in their use of substrate [77–79].

### 3.5. Further Lipid Mediator Research

There are some important questions that remain unanswered in this field, such as “are there potentially deleterious health effects of decreasing the tissue n-6 to n-3 PUFA ratio?” The associations between PUFA content of phospholipids and lipid mediators reveal some possible problems. For example, decreased AA-derived lipoxin-A_4_ biosynthesis occurs with n-3 PUFA dietary supplementation [9,80,81]. Effects such as this may not always be beneficial for health as, for example, decreased lipoxin A_4_ is associated with severe human asthma [82]. The influence of DHA and EPA dietary supplements on asthmatics is not known. To address this, n-3 dietary trials should quantify all lipid mediator substrates within both diet and phospholipids of immune and vascular cells, in contrast to reporting the n-6 to n-3 PUFA ratio [83,84]. Additionally, the tissue lipid mediator profile should be assessed, in contrast to sporadic mediators in previous dietary trials [9,62]. A comprehensive profile, rather than isolated lipid mediators, is important because the lipid mediator network can function in antagonistic, synergistic, or additive manners. Having identified interactions among lipid mediators and their substrates, future research should then investigate the possible causes and interventional targets of such relationships.

Another important question is “whether n-3 PUFA supplementation ameliorates inflammation during human and animal disease because of increased biosynthesis of resolving lipid mediators from n-3 PUFA or decreased proinflammatory lipid mediator biosynthesis from n-6 PUFA?” It was reported that decreasing the tissue n-6 to n-3 PUFA ratio increases the biosynthesis of resolving type lipid mediators from n-3 PUFA and decreases the biosynthesis of proinflammatory lipid mediators from n-6 PUFA [80,81], but comparative health responses between low n-6 PUFA and high n-3 PUFA consumption are not clear. This question could be initially addressed in carefully designed dietary trials that measure phospholipid PUFA composition and lipid mediator biosynthesis of tissue.

The final question that this body of literature raises is “under which circumstances should inflammation be manipulated by dietary n-3 PUFA?” Inflammation is an essential component of mammalian physiology and exists to facilitate restoration of homeostasis in tissue. Anti-inflammatory effects of dietary PUFA may, therefore, have adverse consequences in disease recovery. This has in fact, been demonstrated with dietary n-3 PUFA supplementation in murine models of gastrointestinal inflammation [85]. This does not imply that treatment of uncontrolled inflammation is contraindicated, but rather, that manipulation of lipid mediator biosynthesis likely has multiple outcomes and that some outcomes can be undesirable. Further evidence of this is the discovery that selective COX2 inhibitors affect vasoactive prostaglandin biosynthesis and increase risk of thromboembolic disease in humans [86,87].

## 4. Delivery and Utilization of Dietary PUFA for Phospholipid Biosynthesis

A better understanding of critical regulatory steps that impact phospholipid biosynthesis from dietary PUFA will provide insight into the variable success of dietary manipulation of inflammatory conditions. For example, though phospholipid biosynthesis sometimes displays a preference for absorbed PUFA, not all dietary PUFA will be used for this purpose. Also, enzymes which synthesize phospholipids are influenced by disease and physiological states, such as reproduction. Additionally, PUFA may arise at sites of phospholipid biosynthesis from body stores, such as adipose tissue, and displace dietary PUFA as substrate.

### 4.1. Digestion and Absorption of Dietary PUFA

PUFA are ingested in several forms, including non-esterified fatty acid, triacylglycerol, and phospholipids. Ester bonds of ingested lipids are hydrolyzed by pancreatic lipase to produce predominantly non-esterified fatty acids and monoacylglycerol [10]. This is the primary digestive process for monogastric species, such as humans and swine and is not currently known to affect PUFA supply. However, PUFA from which lipid mediators are derived are very susceptible to non-enzymatic oxidation. For example, the anti-inflammatory effect of n-3 ingestion is increased if co-ingested with antioxidants [30], although it is not clear if the antioxidant effect occurs prior to n-3 absorption, or after, as demonstrated in other models [88]. Multi-gastric species such as ruminants experience an additional digestive process which, in contrast to lipase hydrolysis, has been demonstrated to affect dietary PUFA content [11]. This is referred to as biohydrogenation and describes hydrogenation of PUFA by the micro-flora of the fore-stomachs. Biohydrogenation reduces the efficiency of absorption of some PUFA, particularly those present in the diet at low concentrations such as EPA and DHA. For example, *in vivo* goat models of biohydrogenation demonstrated transfer of only 3.5% to 7.6% of dietary EPA or DHA into milk [89]. Apparently this is sufficient to induce changes in tissue phospholipid composition, possibly because the quantity of absorbed n-3 PUFA is large relative to pre-supplementation tissue and milk levels [22].

The absorption process for available PUFA is similar across all domestic animal species and humans and occurs through the intestinal lymphatic system and the venous side of the vascular system [10]. In this way, absorbed PUFA are protected from hepatic metabolism in the first pass through the circulatory system, and immediately available for biosynthesis of phospholipids in organs and cells critical in determining inflammatory responses, such as bone marrow, peripheral leukocytes, and vascular tissue [10,12].

### 4.2. Phospholipid Substrate from Non-Dietary PUFA

**Figure 3:**
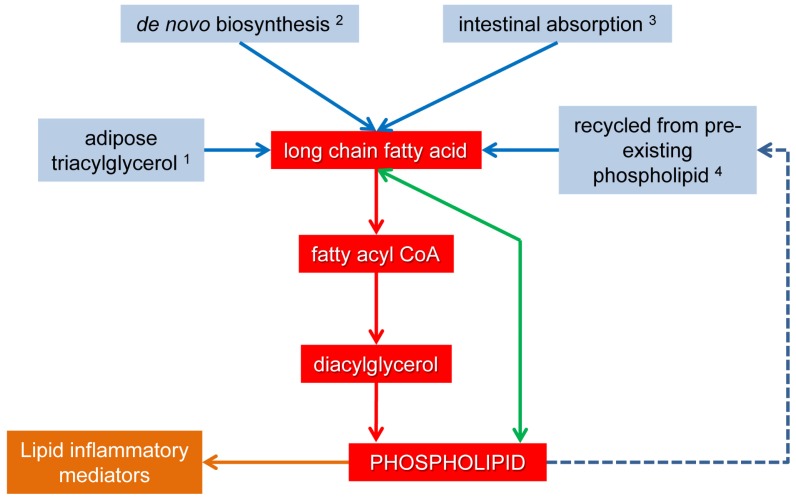
The utilization of long chain fatty acids for *de novo* phospholipid biosynthesis (Kennedy pathway, **red arrows and boxes**), post-synthetic modification of phospholipids by reacylation (Lands’ cycle, **green arrow**), and the metabolism of phospholipids to oxidized lipid mediators of inflammation (**orange arrow and box**) in critical cells during inflammatory responses, such as vascular endothelial cells and mononuclear leukocytes. Origins of fatty acid substrate addressed in this review are (**solid blue arrows and boxes**) adipose triacylglycerol-derived fatty acid ^1^, fatty acid synthesized *de novo*^2^, recently absorbed dietary fatty acid ^3^, and phospholipid-derived fatty acid, cleaved by phospholipases then recycled for phospholipid biosynthesis ^4^ (**broken blue arrow**).

There are several important sources of non-dietary PUFA that confound the influence of dietary PUFA on inflammation (Figure 3) [90]. These include PUFA synthesized *de novo* or stored in sites such as adipose tissue. The relative efficiencies of n-3 and n-6 PUFA *de novo* biosynthesis are controversial. For example, *de novo* biosynthesis of EPA and DHA from ALA has been described as modest [91] but there is evidence that humans have greater capacity for this than *de novo* biosynthesis of AA from LA [92]. Therefore *de novo* biosynthesis of some n-3 PUFA cannot be discounted as having potential health benefits if this results in increased tissue content of DHA and EPA derived pro-resolving type lipid mediators.

Adipose-derived PUFA may also be used for phospholipid biosynthesis in inflammatory based cells (Figure 3). This is an abundant substrate for phospholipid synthesis during dietary restriction or large, chronic expenditures of energy because the rate of lipolysis exceeds that of lipogenesis and adipose tissue is mobilized [93]. This occurs, for example, in humans and domestic animals during the periparturient period [94,95] because of energy expenditure associated with pregnancy and lactation and a concomitant deficit in calorie intake. During adipose mobilization, the liver can utilize adipose-derived fatty acid for ketone body biosynthesis, particularly if carbohydrate ingestion or substrates for gluconeogenesis are restricted relative to the requirements for glucose [10]. Alternatively, fatty acid in the liver may be stored as triacylglycerol. In other organs, such as skeletal muscle, dietary and adipose-derived fatty acid can be energy substrates through β-oxidation, or in functional mammary glands, are excreted as milk lipid. Hence when adipose-derived fatty acids are mobilized into circulation, phospholipid biosynthesis must compete against ketogenesis, β-oxidation, and milk-lipid excretion for PUFA substrate. In spite of this competition, there is *in vitro* evidence that suggests adipose-derived fatty acids modify the composition of phospholipids in vascular endothelial tissue and induce inflammation through lipid mediators, and also evidence that this effect can be reversed with n-3 PUFA supplementation [24,74]. This is particularly interesting as there is an exacerbated inflammatory response *in vivo* in some species coincident with adipose mobilization [96]. This suggests that decreasing the n-6 to n-3 PUFA ratio in adipose could potentially improve health during periods of adipose mobilization. Dietary PUFA are incorporated into adipose triacylglycerol by *de novo* triacylglycerol biosynthesis and remodeling through reacylation [97,98]. The rate of *de novo* synthesis is 20% of total triacylglycerol in healthy human adipose tissue after nine-weeks [99]. The kinetics of triacylglycerol reacylation are not reported but since the half-life of all adipose triacylglycerol is 6-months to 2-years [100], it is reasonable to interpret that reacylation of adipose triacylglycerol occurs over several months or years *in vivo*. These data suggest that a long-term change in diet is required to induce change in adipose n-3 PUFA content. Since DHA and EPA are substrate to several inflammation-resolving lipid mediators, long-term ingestion of these, particularly during periods of triacylglycerol synthesis, could be therapeutic in inflammatory conditions associated with mobilization of adipose tissue.

Another potential origin of PUFA for phospholipids is recycled use of fatty acyl chains that have been hydrolyzed from existing phospholipids (Figure 3). Little is known about the role of this pool of fatty acids in biosynthesis of phospholipids. However, it is unlikely that the plasma membrane, the largest PUFA reservoir, has a major role in supplying PUFA for *de novo* phospholipids as the machinery for this are located in the endoplasmic reticulum and mitochondrion [12].

### 4.3. Effects of Disease, Physiological State, and Other Nutrients on Phospholipid Biosynthesis

**Figure 4:**
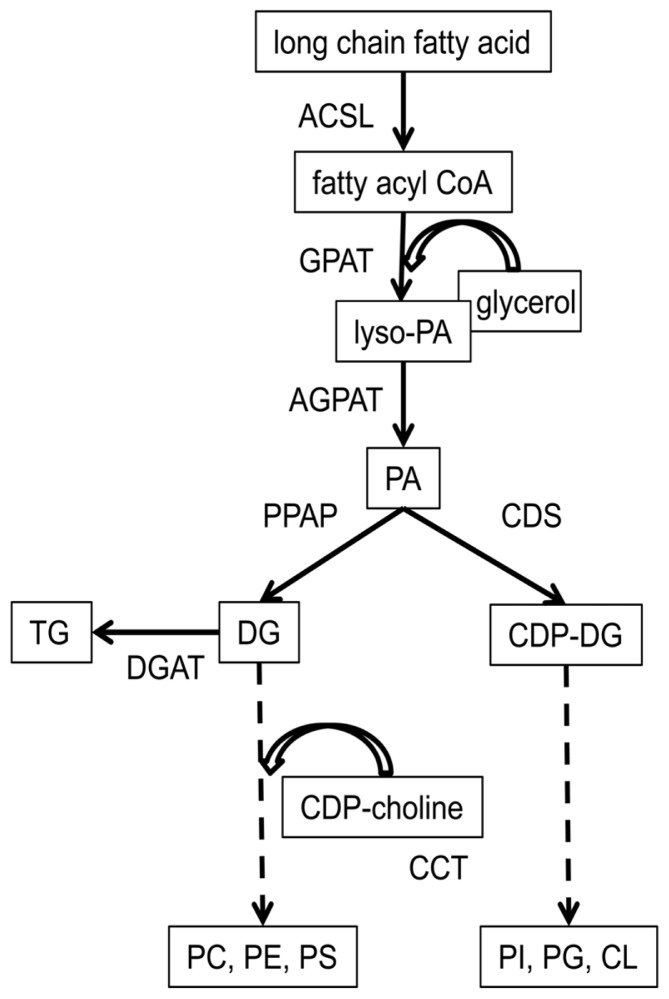
Biosynthetic pathway of major phospholipids in vascular endothelial tissue and mononuclear leukocytes. Metabolites are boxed, enzymes are unboxed. Note that diacylglycerol is a substrate for both triacylglycerol and phospholipids. Key: ACSL: acyl CoA synthase (ligase); AGPAT: 1-acylglycerol-3-phosphoacyltransferase; CDP-choline: cytidine-diphosphate choline; CDS: cytidine-diphosphate diacylglycerol synthase; CL: cardiolipin; CCT: cytidine-triphosphate:phosphocholine cytidylyltransferase; DG: diacylglycerol; DGAT: diacylglycerol acyltransferase; GPAT: glycerol-3-phosphoacyltransferase; PA: phosphatidic acid; PC: phosphocholine; PE: phosphoethanolamine; PG: phosphoglycerol; PI: phosphoinositol; PPAP: phosphatidic acid phosphatase; PS: phosphoserine; TG: triacylglycerol.

*De novo* biosynthesis of phospholipids occurs in what is commonly referred to as the Kennedy pathway [101]. A brief description of this pathway is useful to understand regulation of dietary PUFA incorporation into phospholipids. The first reaction in this pathway combines long-chain fatty acid with CoA and is catalyzed by acyl CoA synthase (ligase) (ACSL, Figure 4). The fatty acyl CoA is then esterified to glycerol at sn-1 on the mitochondria by glycerol-3-phosphoacyltransferase (GPAT) [12]. The second acyltransferase is 1-acylglycerol-3-phosphoacyltransferase (AGPAT). Substrate for AGPAT is transferred from the mitochondria to the endoplasmic reticulum, and acylation occurs at sn-2 of glycerol [12]. Glycerol esterified to fatty acid at sn-1 and sn-2 is known as phosphatidic acid. Phosphatidic acid can be metabolized by phosphatidic acid phosphatase (PPAP or lipin) to synthesize diacylglycerol. This combines with cytidine diphosphate (CDP)-choline to produce phosphocholine, which may be metabolized to phosphoethanolamine or phosphoserine. The enzyme cytidine triphosphate (CTP):phosphocholine cytidylyltransferase (CCT) catalyzes CDP-choline biosynthesis, and CDP-choline:1,2-diacylglycerol cholinephosphotransferase catalyzes the diacylglycerol reaction with CDP-choline [12]. Triacylglycerol is the alternative diacylglycerol product, catalyzed by diacylglycerol acyltransferase (DGAT) [102]. If phosphatidic acid is not utilized for biosynthesis of diacylglycerol, then it can be utilized by CDP-diacylglycerol synthase for synthesis of phosphoinositol, phosphoglycerol, or cardiolipin.

Expression of some phospholipid synthesizing enzymes is influenced by pathogen associated molecular patterns and so is likely influenced by bacterial infection. For example, expression of *ACSL1*, *3* and *4* is increased in RAW 264.7 murine macrophages in response to the TLR-4 agonist, Kdo_2_[103], an endotoxin-like molecule involved in sepsis and mastitis [96]. Monocyte expression of *ACSL1* is also increased in mouse models of type 1 diabetes, and is associated with an inflammatory phenotype and atherosclerosis [104]. Other rodent models indicate that insulin resistance is associated with *AGPAT2* and PPAP1 deficiency [105]. The clinical relevance of these data is not currently understood but transcriptional effects suggest *de novo* biosynthesis of some phospholipids may change during these diseases and therefore affect how dietary PUFA are incorporated into membrane phospholipids in some patients.

Expression of some phospholipid synthesizing enzymes is also affected by physiological state. For example, *ACSL1* hepatic and mammary tissue expressions are increased in dairy cattle during the postpartum period, relative to prepartum [106,107]. *GPAT*, *AGPAT* and *PPAP* expressions are also increased after bovine parturition [93,106,107]. Activities of some enzymes also change with physiological state. Activity of CCT, the rate limiting enzyme in phosphocholine biosynthesis, is regulated by diacylglycerol-induced intracellular translocation to membranes [12]. Translocation results in 1.8-fold increased hepatic activity in the period immediately following parturition in cows [108]. This may also occur when plasma and hepatic non-esterified fatty acid and di- and triacylglycerol are increased, such as in obese humans [109]. In fact, human adipose expression of a *CCT* gene, *Pcyt1a*, is positively correlated with adipose mass [110]. These findings indicate that the capacity for *de novo* biosynthesis of phospholipids may change by reproductive state and adipose mass, and warrant further investigation at all levels of enzyme regulation. Identifying certain physiological states where dietary PUFA is more successful in influencing the PUFA composition of tissue phospholipids could be used to mitigate disease risk.

Glucose has an important influence on biosynthesis of phospholipids. Glucose availability positively affects cyclin-dependent kinase cdc28, which up-regulates triacylglycerol lipase-4 activity [13]. This results in stored fatty acids being available to phospholipids when glucose is abundant and there is a need for new membrane formation and cell proliferation. Loewen (2012) reports that glucose metabolism also influences biosynthesis of phospholipids through phosphatidic acid and the repressive transcription factor Opi1 [13]. Specifically, under conditions of glucose starvation, intracellular pH declines and results in the release of bound Opi1 from phosphatidic acid. The released Opi1 translocates to the nucleus resulting in repression of genes involved in biosynthesis of phospholipids, and thus a conservation of stored fatty acid for more critical needs such as β-oxidation [14]. These data indicate that phospholipid biosynthesis is affected by glucose homeostasis and suggest that dietary PUFA incorporation into phospholipids be investigated with consideration of dietary carbohydrate ingredients.

After *de novo* biosynthesis in the Kennedy pathway, phospholipids are continuously susceptible to post-synthetic deacylation and reacylation at the sn-1 and sn-2 fatty acyl positions (Figure 3) in what is referred to as the Lands’ cycle [111]. This allows absorbed PUFA to quickly change the composition of tissue phospholipids, apparently within several weeks [112]. Manipulation of PUFA in tissue phospholipids by the Lands’ cycle may be particularly successful during bacterial disease as expression of some acyltransferase enzymes is induced by TLR ligands [113]. Further investigation of this effect may identify therapeutic applications for dietary PUFA during these diseases.

### 4.4. Regulation of the Acyl Composition of Phospholipid

Phospholipids have a diverse fatty acid composition. For example Quehenberger *et al.* (2010) measured 31 fatty-acyl variants of phosphocholine and 38 fatty-acyl variants of phosphoethanolamine in human plasma [36]. This diversity is attributable to different combinations of long chain fatty acids at sn-1 and sn-2 of glycerol [36] and is determined by enzyme preference for specific fatty acid substrate. This is important because enzymes in the Kennedy pathway will define the milieu of lipid mediators produced in tissue when they show preference for substrate of lipid mediators. For example, ACSL-3 and −4 show preference for AA and EPA [114]. AGPAT-1, prefers myristic acid (C14:0), palmitic acid, and LA-CoAs, and AGPAT-2, prefers AA-CoA [113]. These enzymes are differentially expressed in models of sepsis and diabetes, and because of their selection of substrate, may partly explain lipid mediator biosynthesis and inflammation during these diseases [103,104]. Substrate selection by enzymes involved in tissue phospholipid biosynthesis has not been evaluated in PUFA dietary studies, but should be considered when interpreting future studies that examine the influence of dietary PUFA on lipid mediator biosynthesis.

Some Lands’ cycle enzymes are selective between fatty acids. For example, the conversion of phosphoethanolamine to phosphocholine by CDP-ethanolamine:1,2-diacylglycerol ethanolaminephosphotransferase favors biosynthesis of sn-1 palmitic acid/sn-2 DHA. This phospholipid then undergoes rapid reacylation with stearic acid (C18:0) at sn-1, and AA, LA, or DHA at sn-2. In fact, the sn-1 stearic acid/sn-2 AA forms of phosphocholine and phosphoethanolamine are usually synthesized by replacement of other acyl chains in existing phospholipids, rather than directly [12]. Therefore the Lands’ cycle can result in rapid and preferential incorporation of DHA and other PUFA into pre-existing phospholipids. This indicates that dietary supplementation with some n-3 PUFA could have especially rapid effects on health, in light of what is known about the effect of dietary n-3 PUFA supplements on inflammation [9].

As mentioned previously, adipose-derived fatty acid can be a major substrate for biosynthesis of phospholipids in some physiological and disease states. In this context, selectivity of fatty acyl incorporation into triacylglycerol could affect the composition of the fatty pool available for phospholipid synthesis during mobilization of adipose. The final step in triacylglycerol synthesis involves DGAT, which adds fatty acyls to the sn-3 position of diacylglycerol. Unfortunately little research has been conducted into substrate selectivity of mammalian DGAT, but in plants it appears that DGAT-2 demonstrates preference for the less abundant, *trans* isomers of PUFA, and there is similar DGAT-1 and −2 specificity for palmitic acid, oleic acid (C18:1), LA, and ALA fatty acyls [102]. In yeast, both DGAT-1 and −2 have broad substrate preferences [115]. In reviews of these enzymes, Yen *et al.* (2008) suggest DGAT-1 prefers oleic over saturated fatty acyls [116], while others indicate DGAT-2 is required for essential fatty acyl (*i.e*., LA and ALA) incorporation into triacylglycerol [117]. In summary, it appears that much work must be done before the selectivity of mammalian DGAT, and their influence on PUFA incorporation into adipose tissue, will be fully understood.

## 5. Conclusions

Human and animal research indicate that for dietary n-6 and n-3 PUFA to modify the pathology of disease, the PUFA content of phospholipids in cells involved in the inflammatory response must reflect the dietary PUFA composition [9,62] and specific phospholipid-derived PUFA must be utilized for biosynthesis of pro-inflammatory or pro-resolving lipid mediators [9]. The roles of lipid mediators in maintenance of health and regulation of inflammation during disease have been introduced in the literature. Whether lipid mediators are pro-inflammatory or pro-resolving is partly determined by the specific PUFA from which the lipid mediator is derived. There are trends in the literature supporting a role for n-3 PUFA in control and prevention of inflammation during disease but it is also important to recognize that some n-6 PUFA are essential nutrients and produce pro- and anti-inflammatory lipid mediators. A number of clinical trials with patients supplemented with fish-oil showed no clear health benefit, and could be explained by factors that affect dietary PUFA incorporation into phospholipids. In conclusion, future studies of the impact of dietary PUFA on tissue phospholipid biosynthesis should examine digestive processes, competition for absorbed PUFA by other physiological processes, supply of PUFA from sources other than diet, obesity and reproductive state, glucose homeostasis, and selectivity of phospholipid synthesizing enzymes for fatty acids. Such research will improve knowledge of phospholipid biosynthesis in humans and domestic animals. Complementary studies that measure inflammatory outcomes and lipid mediator biosynthesis should reveal the true health benefit or cost of n-3 PUFA dietary supplements.
